# A novel model to culture cells from giant cell tumor of bone using three‐dimensional (3D) polycaprolactone scaffold

**DOI:** 10.1002/elsc.202100020

**Published:** 2021-06-29

**Authors:** Eréndira Estrada‐Villaseñor, Margarita Valdés‐Flores, Abelardo Meneses‐García, Phaedra Silva‐Bermudez, Raul Pichardo‐Bahena, Pedro Ostoa‐Saloma, Gabriela Mercado‐Celis, Ernesto D. Delgado‐Cedillo, Anell Olivos‐Meza, Carlos Landa‐Solís

**Affiliations:** ^1^ Pathology Service National Institute of Rehabilitation Luis Guillermo Ibarra Ibarra Mexico City Mexico; ^2^ Genetics Laboratory National Institute of Rehabilitation Luis Guillermo Ibarra Ibarra Mexico City Mexico; ^3^ Pathology Service National Cancer Institute Mexico City Mexico; ^4^ Tissue Engineering and Cell Therapy Unit National Institute of Rehabilitation Luis Guillermo Ibarra Ibarra Mexico City Mexico; ^5^ Department of Immunology Institute of Biomedical Research National Autonomous University of Mexico Mexico City Mexico; ^6^ Laboratory of Clinical Genomics Division of Graduate Studies and Research Faculty of Odontology National Autonomous University of Mexico Mexico City Mexico; ^7^ Bone Tumors Service National Institute of Rehabilitation Luis Guillermo Ibarra Ibarra Mexico City Mexico; ^8^ Sports Orthopedics and Arthroscopy National Institute of Rehabilitation Luis Guillermo Ibarra Ibarra Mexico City Mexico

**Keywords:** 3D PCL scaffold, 3D tumor models, giant cell tumor of bone

## Abstract

Two‐dimensional (2D) culture of cells from giant cell tumor of bone (GCTB) is affected by loss of the multinucleated giant cells in subsequent passages. Therefore, there is limited time to study GCTB with all its histological components in 2D culture. Here, we explored the possibility of culturing GCTB cells on a polycaprolactone (PCL)‐printed scaffold. We also evaluated the viability of the cultured cells and their adherence to the PCL scaffold at day 14 days using immunofluorescence analysis with calcein, vinculin, and phalloidin. Using the histological technique with hematoxylin and eosin staining, we observed all the histological components of GCTB in this 3D model. Immunohistochemical assays with cathepsin K, p63, and receptor activator of nuclear factor (NF)‐κB ligand (RANKL) yielded positive results in this construct, which allowed us to confirm that the seeded cells maintained the expression of GCTB markers. Based on these findings, we concluded that the PCL scaffold is an efficient model to culture GCTB cells, and the cell viability and adherence to the scaffold can be preserved for up to 14 days. Moreover, this model can also be used in subsequent studies to assess in vitro cell–cell interactions and antineoplastic efficacy of certain agents to establish a treatment against GCTB.

**Abbreviations**
GCTB: giant cell tumor of bone
2D: two dimensional
3D: three dimensional
PCL: polycaprolactone

Giant Cell Tumor of Bone (GCTB) is a primary bone tumor and is classified as an intermediate, locally aggressive, but rarely metastasizing tumor. GCTB comprises three types of cells: (1) multinucleated osteoclast‐type giant cells, (2) mononuclear stromal cells, and (3) monocyte‐like cells. Multinucleated giant cells of this tumor are capable of bone resorption and osteolysis. Stromal cells secrete cytokines and factors essential for osteoclast differentiation and also secrete cytokines that attract monocytes by chemotaxis [1, 2]. Stromal cells are considered the proliferative and neoplastic component of GCTB, and multinucleated giant cells along with monocytes are considered the reactive components [2]. Therefore, GCTB is a tumor prototype wherein cell‐cell interactions are essential for differentiation of its cellular components and consequently its progression.

Two‐dimensional (2D) in vitro models have been used to clarify histogenesis, composition, and biological behavior of GCTB and to compare its therapeutic response of GCTB [3, 4, 5]. However, in 2D cultures, cells interact only with their adjacent cells as there are no other cells or extracellular matrix present above or below them. Another characteristic of 2D cultures is that cells are exposed homogeneously to nutrients and drugs in the medium.

Conversely, three‐dimensional (3D) culture models more accurately recreate the in vivo microenvironment and cell behavior conditions than 2D models. 3D models show cell–cell interactions, access of cells to nutrients, and protein expression different from those observed in 2D models. Additionally, 3D‐printed scaffolds which were initially used in tissue engineering to support bone regeneration, are now used to study the behavior and protein expression of tumor cells and to evaluate the efficacy of antineoplastic drugs [6]. Among these, polycaprolactone (PCL) scaffolds provide a surface that allows adherence and growth of neoplastic cells such as those from osteosarcoma, breast cancer, or Ewing sarcoma [7, 8, 9].

For GCTB, only 2D models have been used in vitro, which is limited by loss of multinucleated giant cells in subsequent passages. Once the primary culture becomes confluent and passaging is necessary, each passage leads to a gradual loss of multinucleated giant cells. With multiple passages, the end result is a monolayer of fibroblast‐like cells, which are morphologically distinct from the original histological components observed in GCTB [3, 4, 5]. Therefore, there is only a limited time frame to study a GCTB with all its histological components in 2D culture. This led us to explore a novel culture model for GCTB cells, using a 3D‐printed PCL scaffold, wherein not only can all the histological components be observed but the 3D arrangement of neoplastic cells can also be recreated.

The main aims of this study were as follows: (1) to explore the possibility of culturing GCTB cells on a PCL scaffold, (2) to evaluate viability of the cultured cells and their adherence at day 14, (3) to determine whether it is possible to observe all the histological components of GCTB using this 3D model, and (4) to explore if this model can be used to study GCTB cell culture using other techniques.

The protocol used in this study was approved by the Research and Ethics Committee of our Institution. All patients provided informed consent to participate in this study. Tissue samples were collected from the patients upon diagnosis of GCTB during surgery but without previous treatment.

**PRACTICAL APPLICATION**

Three‐dimensional (3D) polycaprolactone printed scaffolds can be used to culture and analyze cells from giant cell tumor of bone (GCTB), with the possibility of using immunohistochemistry. Future research using this model will allow the study of cell‐cell interactions and cell‐matrix interactions in GCTB. Additionally, comparative studies on cell proliferation and protein expression can be conducted between this 3D model and two‐dimensional (2D) models along with drug tests to identify potential treatment candidates. The 3D disposition of the cells in this model would allow study differential protein expression in cells based on their tumor location (periphery or central portion), and mechanisms of growth and invasion to adjacent structures. This would further allow research of the type of cells that populate first the adjacent structures of the tumor and the underlying mechanisms by which the other histological components of the tumor arrive at the periphery.

A sterile technique was employed to obtain the tumor tissue samples for culture it. We first determined the number of cells present in 5 mm^3^ of three tumor sample each and calculated the average number of cells; based on this, we assessed the number of cells seeded when using 5mm^3^ of tumor tissue. For this purpose, 5 mm^3^ of tumor tissue was mechanically segregated into small pieces using sterile scissors and a scalpel, and thereafter, enzymatically digested using 0.3% collagenase (Worthington) and shaken at 200 rpm for 2 h at 37°C. The cells were filtered through a 100 μ cell filter and subsequently quantified using the Neubauer chamber. The average number of cells in 5 mm^3^ of tumor tissue sample was 1,745,333.

We used the REGEMAT 3D Bioprinting system (Universidad de Granada, Spain) to prepare the scaffold using a pellet of PCL. The scaffold was printed with the following specifications: pore size of 850 μ, total of seven layers with strand thickness of 200 μm, flow rate of 1, without solid perimeter, printing angle of 90^o^, without a solid base, and forming a 5 × 5 mm^3^ cube on each face. For sterilization of the PCL scaffold, we were cautious that the melting temperature of PCL (58−60°C) should not be reached and used a SterradNx medical sterilizer. Scaffolds were subjected to one cycle of sterilization at 30°C. This is a chemical method of sterilization that uses plasma/hydrogen peroxide vapor. However, there are other chemical methods that can be used for this process, such as ethylene oxide [10].

For cell seeding, 5 mm^3^ of tumor tissue sample was used for each PCL scaffold. GCTB tissue was mechanically homogenized, using in addition to scissors and a scalpel, medium to preserve cell viability during this process. Once a smooth consistency was obtained, the suspension was collected in a 2 mL pipette. Next, the tip of the pipette was placed in direct contact with the scaffold, and its content was slowly and carefully released, yielding a construct (Figure 1A). The constructs were placed in six well culture plates with Dulbecco's modified Eagle's medium (DMEM) supplemented with 10% fetal bovine serum and 1% penicillin/streptomycin. These were then stored in a humidified incubator at 37°C with 5% carbon dioxide (CO_2_) atmosphere. The medium was replaced every 3 days until day 14.

**FIGURE 1:**
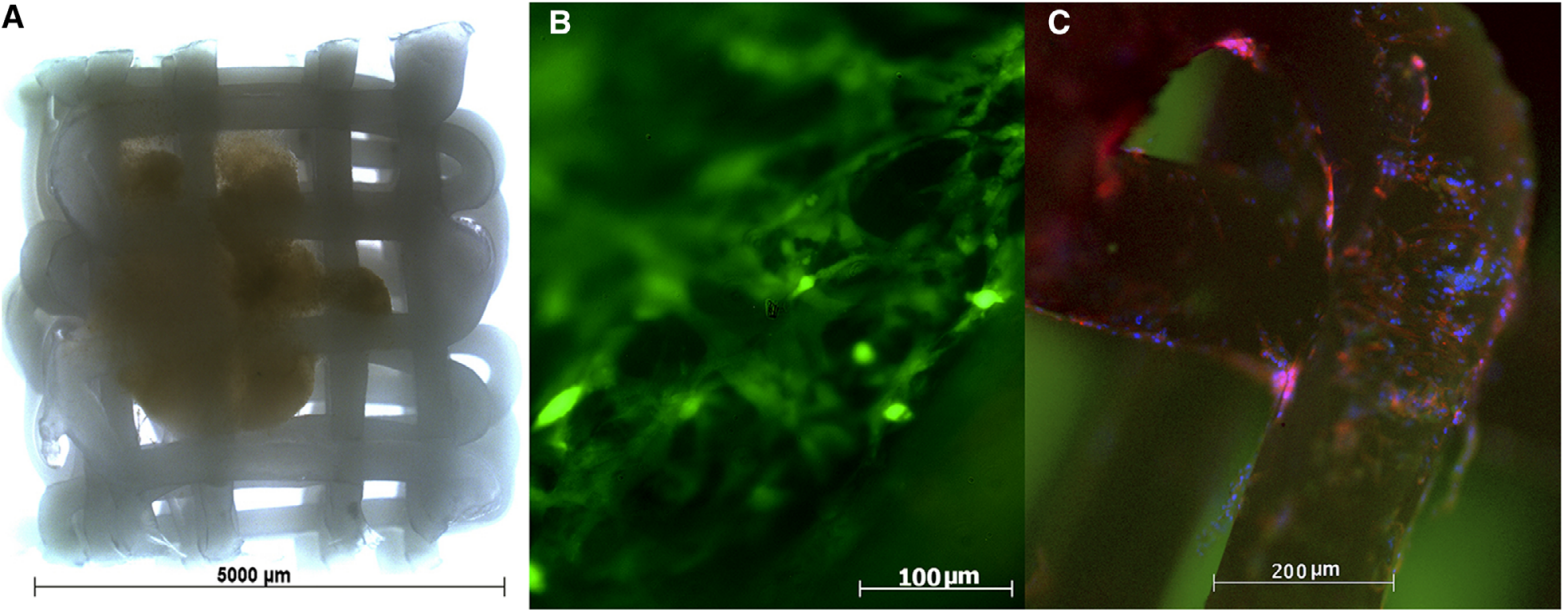
(A) Photomicrograph with stereoscopic microscope of initially giant cell tumor of bone (GCTB) seeded cells on polycaprolactone (PCL) scaffold. (B) Immunofluorescence for calcein in the periphery of PCL scaffolds with GCTB cells after 14 d of culture. Viable cells are marked in green (40x). (C) Immunofluorescence for 4’,6‐diamidino‐2‐phenylindole (DAPI), vinclulin, and phalloidin in the periphery of the PCL scaffolds with GCTB cells after 14 d of culture. The nuclei are arranged in groups and individually. Cells are positive for vinculin and palloidin (20x)

Two weeks after the GCTB cells were seeded in the PCL scaffold, the construct was stained with calcein (Cayman chemical) to determine the cell viability on the scaffold. For this, the construct was incubated in a six well plate (Corning) for 30 min with DMEM plus 0.2 mg/mL of calcein in an incubator at 37°C supplied with 5% CO_2_. Thereafter, the culture medium with calcein was replaced with complete culture medium (DMEM, 10% serum and 1% antibiotic/antimycotic). Images were immediately recorded using an inverted microscope with an ultraviolet (UV) lamp (Carl Zeiss; Axiovert 25; software image vision 4.8.2). The GCTB cells seeded on the PCL scaffold were found to be positive to calcein, which confirmed their viability (Figure 1B). Calcein positivity was observed in the cytoplasm of the cells attached to the PCL fibers as well as the cells that grew between the PCL fibers.

To determine the extent of cell adhesion on the scaffold, 2 weeks after the GCTB cells were seeded, the construct was stained using Actin Cytoskeleton and Focal Adhesion Staining Kit (Merck Millipore). The construct was fixed, incubated with primary and secondary antibodies, and washed according to the manufacturer's instructions. The images were immediately recorded using a pyramidal microscope attached with a UV lamp (Carl Zeiss; Imager A1 AX10; software image vision 4.8.2). The cultured cells were positive for vinculin and phalloidin. Further, upon staining with DAPI, the nuclei in the fibers were observed to be arranged in groups and also present individually (Figure 1C). Based on these results, we confirmed cell adherence to the PCL fibers as well as occurrence of cell‐cell and cell‐matrix interactions.

Although fluorescence was observed in both the central and peripheral portions of the scaffold, the photomicrographs for cell viability (Figure 1B) and cell adhesion (Figure 1C), were captured in the periphery of the scaffold, owing to the high cell density in the central portion of the scaffold that did not allow to appreciate the fluorescence in a clear way on this zone. From the observations in Figures 1B and 1C, we concluded the following: (1) the cells that grew out from the original tumor were viable and could adhere to the PCL scaffold, and (2) most of the cells that predominate in this peripheral area of the scaffold at 14 days were mononucleated and most likely correspond to the stromal cells or monocytes of the GCTB. These findings are important for future research using this 3D model as they would allow further investigation of how the tumor grows and invades adjacent tissues. Finally, with this 3D model, we could assess the type of cells that primarily populate adjacent tissues of the tumor and the underlying mechanisms through which the other histological components of the tumor arrive at the periphery.

For hematoxylin and eosin staining, the constructs were fixed using 10% neutral‐buffered formalin for 1 h, followed by dehydration in graded alcohol solutions. Thereafter, they were placed in 50% alcohol‐50% xylene for 30 min, followed by two changes of liquid paraffin (1 h each) at 55°C. Finally, they were embedded in paraffin blocks. A microtome (model RM2165, Leica Microsystems) was used to cut the paraffin blocks into 5‐μm thick sections. The slides were deparaffinized and hydrated in water, stained with hematoxylin for 5 min and eosin for 1 min, dehydrated in graded alcohol solutions, cleared with xylene, and then mounted. Histological analysis of the constructs using hematoxylin and eosin staining revealed the presence of viable neoplastic cells and all histological components of GCTB in the scaffolds, including the multinucleated osteoclast‐type giant cells, stromal cells with spindled nuclei, and monocytes. With this technique, we observed morphological preservation and verified that there was no loss of multinucleated giant cells, typically observed in 2D cultures during subsequent passaging (Figure 2A). A possible explanation for the absence of multinucleated giant cell loss could be that in the PCL scaffold, 3D arrangement of the cells allows greater interaction among cells, accompanied by stronger and longer‐lasting effect of the cytokines and differentiating factors secreted by stromal cells, which are essential for osteoclast differentiation. It could also be possible that in this 3D model, the cytokines remain localized in the scaffold microenvironment, thereby favoring the differentiation and permanence of multinucleated giant cells instead of being dispersed as could happen in 2D cultures.

**FIGURE 2:**
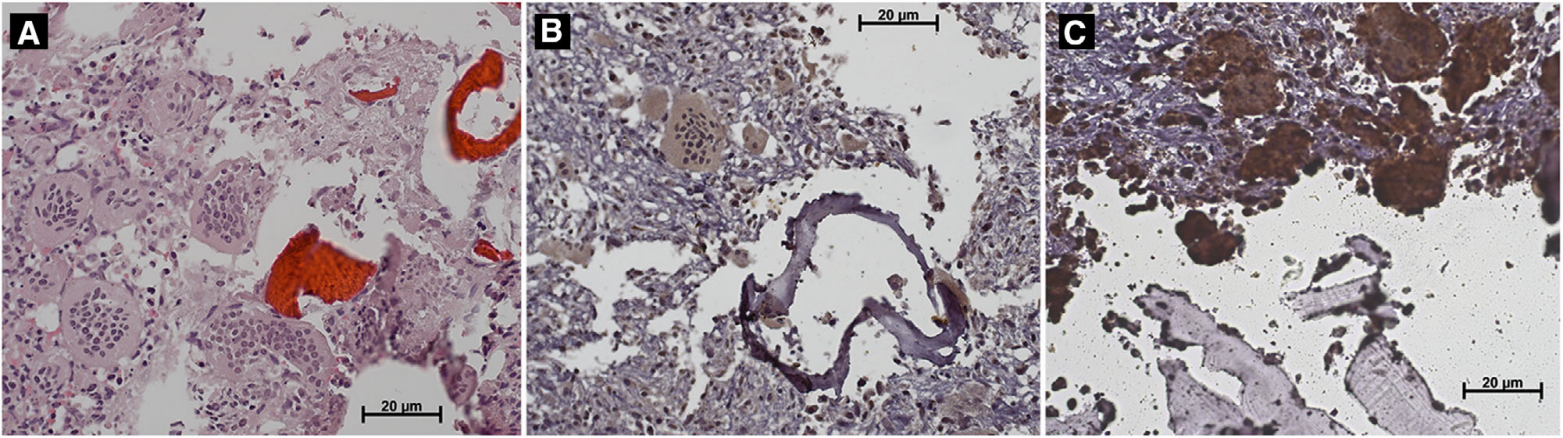
(A) Hematoxylin and eosin staining of polycaprolactone (PCL) scaffolds with giant cell tumor of bone (GCTB) cells (40x). The PCL fibers are shown in reddish‐orange color. The PCL fibers are alternating with multinucleated giant cells, stromal cells, and monocytes Polycaprolactone fibers, multinucleated giant cells. (B) Immunostaining for p63 showing positivity in the nuclei of some stromal cells (40x). (C) Immunostaining for cathepsin K showing intense positivity in the cytoplasm of multinucleated giant cells (40x)

We next performed immunohistochemistry analysis to determine whether this model could be useful to study GCTB cell culture with other techniques and confirmed that the observed cells were not only morphologically GCTB cells but also expressed typical GCTB markers, such as p63, cathepsin K and RANKL [11, 12, 13]. The primary antibodies used included mouse monoclonal antiP63 (1:200, Sta Cruz Biothechnology) mouse monoclonal anti‐RANKL (1:150 Sta Cruz Biothechnology), and mouse monoclonal anti‐cathepsin K (1:200 Sta Cruz Biotechnology). The Mouse/Rabbit ImmunoDetector DAB Detection System (Bio SB) was used. Instructions were followed according to the manufacturer. The positive control included sections of GCTB from the archives of the Anatomic Pathology service. The immunohistochemistry assays for p63 and RANKL yielded positive results in stromal cells, whereas the assay for cathepsin K yielded positive result in the cytoplasm of multinucleated giant cells (Figure 2B and Figure 2C). We therefore confirmed the cultivation of GCTB cells in the PCL scaffold and also the possibility of using other techniques, such as immunohistochemistry, in the study of GCTB cultured cells, in the PCL scaffold.

The results of this study demonstrate that the GCTB cells can be cultured in 3D printed scaffolds of PCL for 14 days, conserving their viability and adherence and without the loss of multinucleated giant cell, which is typically observed in conventional 2D culture systems. Further, this model offers the possibility of using other techniques, such as immunohistochemistry, to investigate GCTB cells. However, although PCL scaffolds can simulate bone porosity and stiffness, a more bone‐like environment can be achieved using 3D PCL scaffolds coated with calcium phosphate/hydroxyapatite material or printed with type 1 collagen materials. This way, the extracellular matrix that would be interacting with the seeded cells on the scaffold would be more like the bone. Therefore, further studies could be done using these coated scaffolds to elucidate cell‐cell and cell‐extracellular matrix interactions in a more representative microenvironment.

In conclusion, to the best of our knowledge, this study demonstrated for the first time that 3D printed PCL scaffolds can be used for culturing GCTB cells, as they preserve the viability, adherence, morphology, and all the histological components of this tumor. This model also facilitates the use of techniques such as immunohistochemistry in the study of GCTB cells. Therefore, this model could be a better option than 2D models to study GCTB cells, to learn about cell‐cell interactions and to test new therapeutic drugs against GCTB.

## CONFLICT OF INTEREST

The authors have declared no conflict of interest.

## Data Availability

The data that support the findings of this study are available from the corresponding author upon reasonable request.
